# Managing Fear During Pandemics: Risks and Opportunities

**DOI:** 10.1177/17456916231178720

**Published:** 2023-06-26

**Authors:** Gaëtan Mertens, Iris M. Engelhard, Derek M. Novacek, Richard J. McNally

**Affiliations:** 1 Department of Medical and Clinical Psychology, Tilburg University; 2 Department of Clinical Psychology, Utrecht University; 3 Desert Pacific Mental Illness Research, Education, and Clinical Center, VA Greater Los Angeles Healthcare System, Los Angeles, CA; 4 Department of Psychiatry and Biobehavioral Sciences, University of California, Los Angeles; 5 Department of Psychology, Harvard University

**Keywords:** fear, COVID-19, pandemic, health behavior

## Abstract

Fear is an emotion triggered by the perception of danger and motivates safety behaviors. Within the context of the COVID-19 pandemic, there were ample danger cues (e.g., images of patients on ventilators) and a high need for people to use appropriate safety behaviors (e.g., social distancing). Given this central role of fear within the context of a pandemic, it is important to review some of the emerging findings and lessons learned during the COVID-19 pandemic and their implications for managing fear. We highlight factors that determine fear (i.e., proximity, predictability, and controllability) and review several adaptive and maladaptive consequences of fear of COVID-19 (e.g., following governmental health policies and panic buying). Finally, we provide directions for future research and make policy recommendations that can promote adequate health behaviors and limit the negative consequences of fear during pandemics.

Fear is triggered by the perception of imminent danger and serves the adaptive function of mobilizing energy for coping with a threat. Nonetheless, when fear is chronic, excessive, or both in relation to the threat, it can evoke substantial distress that interferes with daily life. The recent COVID-19 pandemic provided ample triggers for fear (e.g., public-health restrictions; financial setbacks; images of people on ventilators; daily updates of numbers of new cases; and experiencing symptoms of the common cold, flu, or seasonal allergies; Asmundson & Taylor, 2020; Mertens et al., 2020). Indeed, fear in the context of the COVID-19 pandemic is a multifaceted construct, with concerns relating to health, supply shortages, xenophobia, and socioeconomic worries (Mertens et al., 2020; Taylor et al., 2020a). Nonetheless, studies have identified the central focus of COVID-19 fear to be the perceived threat of being infected by the SARS-CoV-2-virus and the potentially dangerous health-related consequences of such an infection, both for oneself and for family and friends (Mertens et al., 2021; Taylor et al., 2020b). This fear of COVID-19 is distinguishable from broader anxiety-related constructs, such as intolerance of uncertainty and health anxiety (Mertens et al., 2021; Taylor, Landry, Paluszek, et al., 2020). Given that threat perception and fear can motivate health protective behaviors, according to the health-belief model and protection-motivation theory (Rogers, 1975; Rosenstock, 1974), it is important to further examine this emotion within the context of the COVID-19 pandemic.

Several factors influence the intensity of fear. Proximal threat (e.g., being held at gunpoint) often triggers intense fear or terror, whereas distal threat (e.g., having to give an important presentation next week) often fosters anxious foreboding (Grillon, 2008). Furthermore, unpredictable threats are usually more distressing than predictable ones (Shankman et al., 2011). Finally, the possibility of evading a threat or controlling its impact diminishes distress (Hartley et al., 2014). Subjective appraisals of proximity, predictability, and controllability determine fear, hence underscoring the importance of an individual’s self-report of such appraisals. Consequently, self-report questionnaires were recently developed to measure subjectively experienced fear within the context of the COVID-19 pandemic, such as the Fear of COVID-19 Scale (Ahorsu et al., 2020) and the Fear of the Coronavirus Questionnaire (Mertens et al., 2020). Responses on these questionnaires predict several adaptive and maladaptive consequences of fear of COVID-19, as we review below.

Fear is an evolutionary evolved emotion and has adaptive value to increase an organism’s chances of survival by causing the organism to avoid or adequately respond to potential threats (i.e., the “fight-or-flight” response; Marks & Nesse, 1994). Nonetheless, when fear is evoked by mild threats, such as pictures of snakes and spiders, it can have maladaptive costs such as losing valuable energy and opportunities. Similarly, during the COVID-19 pandemic, high levels of fear could motivate people to take adequate safety precautions but could also cause distress or impairment in important areas of functioning (e.g., avoiding seeing friends and family, even when the risk of infection is low—such as when meeting outside). On the other hand, low levels of fear can minimize distress but can also expose people to risks, such as infection (e.g., contracted during lockdown parties). Hence, fear should be adjusted to risks in the environment. Both excessively high and low levels of fear have their costs. Here, we will review the emerged evidence of the adaptive and maladaptive consequences of fear during the COVID-19 pandemic.

## Adaptive and Maladaptive Consequences of Fear Within the COVID-19 Pandemic

### The adaptive function of fear during the COVID-19 pandemic

#### Adherence to public-health measures

Studies have shown that increased fear of COVID-19 was related to taking precautions to reduce the probability of infection, notably better adherence to governmental health policies, such as adhering to social distancing, limiting social contacts, practicing hand hygiene, and wearing face masks. For example, in an international community sample, Harper et al. (2020) found that fear of COVID-19 was related to more handwashing and social distancing. Other cross-sectional and longitudinal studies have found similar results (e.g., Broomell et al., 2020; Knowles & Olatunji, 2021; Winter et al., 2020).

#### Vaccine uptake

Given that many studies have shown that COVID-19 vaccines have demonstrable effectiveness to prevent severe symptoms and death (Zheng et al., 2022), vaccine uptake is adaptive within the context of the COVID-19 pandemic. Several studies have found that fear of COVID-19 is positively related to COVID-19 vaccine uptake. For example, in a longitudinal sample, both fear of COVID-19 measured concurrently and 14 months earlier (i.e., at the beginning of the pandemic) predicted COVID-19 vaccination uptake (Mertens et al., 2022). Multiple studies have replicated this pattern (e.g., Bendau et al., 2021; Vollmann & Salewski, 2021).

#### Protection against long COVID

Furthermore, fear of COVID-19 likely protects against *post-COVID-19 syndrome*, or long COVID. Long COVID is characterized by a range of persistent health problems beyond the initial illness. These include fatigue, shortness of breath, muscle weakness, memory problems, and other cognitive dysfunctions. Given that vaccines offer protection against the development of long COVID (Al-Aly et al., 2022), fear of COVID-19 likely provides an indirect protective effect against long COVID through the higher vaccine uptake described in the previous section. However, to our knowledge, there are no studies yet that have directly related individual-level fear of COVID-19 to reduced risk for long COVID.

### The maladaptive consequences of fear during the COVID-19 pandemic

#### Mental-health problems

Because of the stress and uncertainty created by the COVID-19 pandemic (e.g., loss of routines, income, and social contacts), it is unsurprising that the pandemic had a negative influence on people’s mental health, particularly for those who experienced increased fear of COVID-19. Indeed, studies have found that fear of COVID-19 is positively correlated with mental-health problems and stress symptoms, such as anxiety, depression, stress, and sleep problems (Kim et al., 2022; Siddique et al., 2021; Şimşir et al., 2022). For example, in a meta-analysis by Şimşir et al. (2022), moderate-to-strong correlations were found between the Fear of COVID-19 Scale (see Ahorsu et al., 2020) and anxiety (*r* = .55), stress (*r* = .47), and depression (*r* = .38).

Moreover, the COVID-19 pandemic has also increased clinical levels of anxiety-related symptoms, such as those related to obsessive-compulsive disorder and posttraumatic stress disorder (PTSD; Tanir et al., 2020; Taylor, 2022). For example, health-care workers and individuals who required hospitalization because of COVID-19 were at an elevated risk of experiencing PTSD-related symptoms (Rossi et al., 2020).

However, not everyone is equally likely to suffer from these fear-related mental-health problems. Individuals who are more prone to *threat reactivity* (i.e., a broad term referring to predisposing factors for internalizing symptoms, such as threat appraisals, attentional bias, and intolerance of uncertainty) seem especially likely to develop mental-health problems (Funkhouser et al., 2022).

#### COVID-19 minimization and antivaccination attitudes

Although higher levels of fear of COVID-19 are related to more hygienic behaviors and greater willingness to receive vaccination (see above), lower levels of fear of COVID-19 are related to a belief that the threat of COVID-19 is exaggerated, a view that leads to reduced hygienic behaviors and an increase in antivaccination attitudes (Mertens et al., 2022; Taylor, Landry, Paluszek, et al., 2020). Given the evidence for the effectiveness of good hygiene to prevent infection and of vaccination to prevent severe COVID-19 symptoms (Lio et al., 2021; Tenforde et al., 2021), these consequences are maladaptive.

Additionally, other fears seemingly contribute to vaccination hesitancy as well: for example, fear of needles and fear of harmful side effects (e.g., concerns about fertility; for a review, see Galanis et al., 2021). Hence, different fears may affect an individual’s cost-benefit analysis influencing the decision to get vaccinated against COVID-19.

#### Panic buying and the tragedy of the commons

During the COVID-19 pandemic, there were reports of panic buying of certain goods, such as toilet paper and medication. Studies found a relationship between panic-shopping behaviors and fear of COVID-19 (Taylor et al., 2020a; Taylor, Landry, Rachor, et al., 2020). Interestingly, panic buying exemplifies how fear can have ironic self-fulfilling effects. Because of the perceived risk of shortages, some people engaged in panic buying of large quantities of supplies (Chua et al., 2021), overloading supply chains and (temporarily) creating the dreaded shortages themselves. More generally, this pattern illustrates the so-called “tragedy of the commons,” in which what is good at the level of the individual proves bad at the level of the group. This, in turn, becomes bad for the individual when each member of the group exhibits the behavior (Hardin, 1968). This tragedy-of-the-commons concept can be applied to other problems described here, such as COVID-19 vaccine refusal based on the reasoning that if everyone else is vaccinated, one individual will be protected without getting the vaccine and without being exposed to any of its possible side effects (i.e., fear of vaccination referenced elsewhere). This ultimately results in lower vaccination rates and a failure to reach the levels of immunization required to achieve herd immunity (Savulescu et al., 2021).

#### Xenophobia

Because the initial outbreak of the SARS-CoV-2-virus occurred in the Wuhan province of China, fear of COVID-19 has also fueled xenophobia against people of Asian descent. Indeed, studies have found that fear of COVID-19 was related to more stigmatization and avoidance of foreigners (Taylor, Landry, Rachor, et al., 2020) and people of Asian ethnicity (Lantz & Wenger, 2022). Furthermore, fear of COVID-19 is related to more avoidance of health-care workers because of the higher chance they carry of being infected by the virus (Taylor, Landry, Rachor, et al., 2020).

#### Social inequality

The experience of fear of COVID-19 is not equal among subsets of the population. Racial and ethnic minorities report more fear of COVID-19 compared with Whites (Niño et al., 2021). Racial and ethnic minorities were more likely to view COVID-19 as a threat to individual and population health at various points during the pandemic. These differences likely result from higher COVID-19 infection and mortality rates in racial- and ethnic-minority communities (Feldman & Bassett, 2021), which in turn is probably because these groups often work in essential occupations where social distancing is difficult (e.g., supermarket employees, health-care workers) and have lower vaccination rates (Galanis et al., 2021). In addition, elderly people and women were more likely than younger people and men, respectively, to report high levels of threat and fear of COVID-19 (Niño et al., 2021).

Similarly, many disabled and immunocompromised individuals experience fear related to COVID-19 infection, death, medical rationing, and medical ableism (Al-Rahimi et al., 2021; Andrews et al., 2021; Lund et al., 2020; Reber et al., 2021). These concerns likely cause elevated rates of generalized anxiety disorder and major depressive disorder in individuals with disabilities (Wang et al., 2022). Indeed, fear among disabled and immunocompromised communities may rise as public-health restrictions are lifted. Hence, both low and high fear of COVID-19 and related policies (e.g., loosening restrictions) can exacerbate socioeconomic inequalities and affect specific groups, such as health-care workers and immunocompromised individuals.

## Recommendations for Policy

**Fig. 1.**
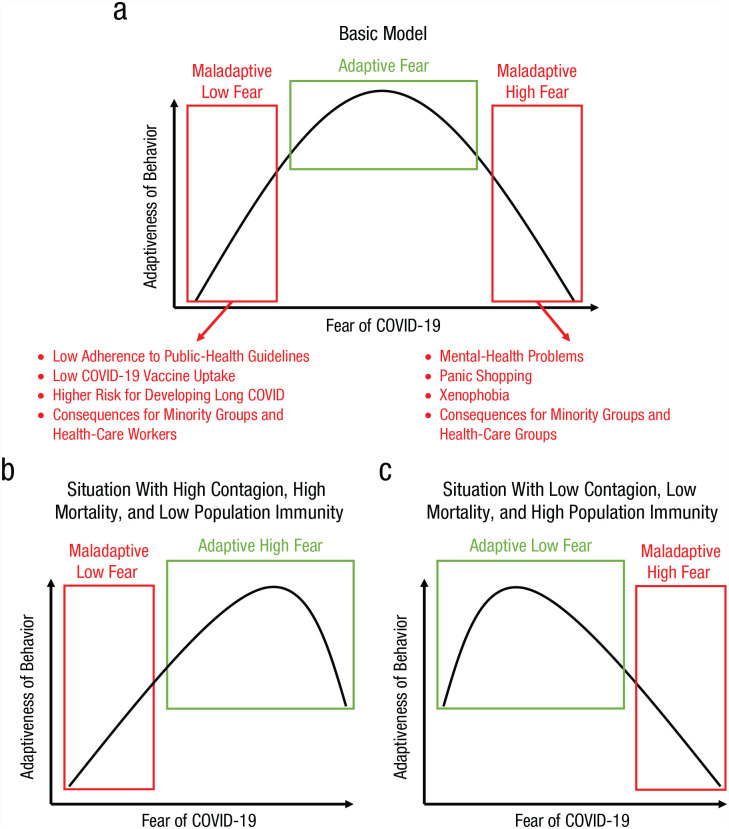
Schematic illustration of the suggested quadratic relation between levels of fear of COVID-19 and adaptiveness of fear-related behaviors (in terms of hygiene, mental-health problems, defensive responses toward minority groups, etc.). In (a), we depict the basic model in which both excessively high and low fear can have adaptive costs. In (b) and (c), we illustrate how adaptiveness of fear levels and its associated behaviors depends on contextual variables, such as the transmission dynamics and mortality rate of the pathogen. Note also that these contextual variables typically change throughout the course of a pandemic such as through the buildup of natural immunity and the availability of more effective vaccines and medication.

Fear during the pandemic is a double-edged sword: It can motivate protective behaviors but can also lead to mental-health problems, risk minimization, vaccination hesitancy, panic buying, xenophobia, and the exacerbation of preexisting socioeconomic inequalities. In other words, there seems to be an optimal point of fear to motivate adaptive behavior, and both excessively low and high levels of fear can have negative consequences (see Fig. 1a). Nonetheless, it should be noted that optimal fear in the context of a pandemic depends on contextual factors, such as the deadliness of the pathogen, the dynamics of transmission, and the availability of effective medication (see Figs. 1b and 1c). Given the central role of fear to motivate behaviors within the context of pandemics, careful consideration is needed to managing fear.

One challenge for policymakers in a pandemic is to motivate people to take adequate precautions against infection. According to the health-belief model and protection-motivation theory, fear is one factor that motivates health behaviors (Rogers, 1975; Rosenstock, 1974). Increasing fear may motivate compliance with containment measures (as we have reviewed previously), but its effect is offset by the undesirable side effects of fear (e.g., distress) and the associated unnecessary safety behaviors (e.g., panic buying). It is important to note that research suggests that threat perceptions and fear motivate health behaviors most often when self-efficacy is high (Ruiter et al., 2014). *Self-efficacy*, within the context of threat, refers to an individual’s appraisal of being able to mitigate a threat (Rogers, 1975).^1^ Therefore, a better course of action than solely increasing fear is to focus on fostering self-efficacy by communicating specifically which behaviors people can adopt to minimize risks for themselves and their social circle (Jørgensen et al., 2021; Peters et al., 2013), such as getting vaccinated, undergoing regular testing, and practicing social isolation when positive for the coronavirus. Additionally, people can become desensitized to repeated threat messages and fear appeals over time (Stevens et al., 2021), further limiting the utility of a solely fear-focused approach in terms of promoting public health.

Another challenge concerns scientific uncertainty regarding the dynamics of a pandemic, which can remain unclear for many months or even years, even though information needs to be shared and public-health decisions must be made. As we indicated, uncertainty tends to increase fear (Grillon, 2008). It may therefore be tempting for health agencies and other actors to minimize uncertainty in their communications. However, having to update initial statements that were communicated with low indications of uncertainty can create resentment and lead people to mistrust science and policies (Petersen et al., 2021). Consequently, we recommend that health agencies and other relevant actors acknowledge uncertainty in their communications, while also providing information that motivates behaviors that are adaptive in the service of public health and that help people to regain a sense of controllability and predictability (i.e., self-efficacy). Moreover, it is important that health agencies provide a realistic image of science and its developing insights, emphasizing that science is characterized by responsiveness to new data, not by timeless truths unmodifiable by new knowledge.

Public-health messaging must also consider issues of equity. As we indicated earlier, marginalized and vulnerable groups have reported experiencing more fear of COVID-19 than other groups. To some extent, this reflects their heightened medical vulnerability (as with, e.g., immunosuppressed groups). However, for other groups, such as racial or ethnic minority groups, this could also reflect biased threat perceptions or unequal access to high-quality health care, which creates social vulnerabilities (Feldman & Bassett, 2021; Stockman et al., 2021). Thus, a two-step approach is likely needed to address disparities between these groups—targeted messaging efforts for minority groups (e.g., translated information on COVID-19 vaccines) and solutions for existing inequalities in health care.

Finally, for those at the high end of the fear distribution, mental-health support may be needed to counter the negative effects of high levels of fear (see Fig. 1). For those who continue to isolate themselves, despite available vaccines and better medications against COVID-19, their patterns of behavior may constitute an anxiety disorder warranting professional treatment (Arora et al., 2020; Arpaci et al., 2020). Without assistance, some individuals may continue to engage in avoidant behavior and social isolation. This behavior is self-reinforcing because it is effective to prevent infection, but it also has significant drawbacks, because it incurs costs such as not seeing friends and family or undertaking other social activities. For those people, psychotherapy could be needed to break the self-reinforcing cycle of avoidance.

## Areas for Future Research

Throughout this review we have noted open questions regarding fear of COVID-19. Here, we will reiterate some important areas for future work for psychological and behavioral science.

A first direction is to investigate how public-health messages during pandemics can be optimally shaped so that they stress the severity of the risks and motivate precautions without inducing excessive fear and uncertainty. Messages emphasizing self-efficacy in protecting oneself are likely more useful than those focusing solely on fear. However, more research is needed to understand what sort of messages work for which people, and under which conditions (Overton et al., 2021). Furthermore, more work is needed to understand how governmental agencies can effectively communicate information about a rapidly changing situation without appearing contradictory or causing unnecessary uncertainty (Petersen et al., 2021).

A second research direction is to improve identification of individuals at heightened risk of developing mental-health problems due to pandemic-related stressors and to determine the most cost-effective ways to mitigate their distress. Conversely, research should also focus more on which people are resilient under the stress of a pandemic (Vos et al., 2021) and which groups seem to thrive (e.g., some reports showed better mental health for some children and adolescents during the COVID-19 lockdown periods; e.g., Soneson et al., 2022). This approach could yield valuable information on how resilience during pandemics can be boosted.

A third research direction is to investigate whether existing clinical protocols, such as exposure-based programs (e.g., Zhang et al., 2020), are effective to help people suffering from clinical levels of fear because of the pandemic.

Finally, a fourth direction is to investigate why minority and marginalized groups are especially adversely affected during a pandemic. Given reports of higher death rates and lower vaccination uptake in these groups during the COVID-19 pandemic (e.g., Feldman & Bassett, 2021), it is imperative to understand how these groups can be reached and supported.

## Summary and Conclusion

The COVID-19 pandemic has produced an abundance of research into the role of fear in predicting different adaptive and maladaptive outcomes. We reviewed emerging evidence that fear can be related to adaptive health behaviors such as social distancing, hygienic behaviors, and vaccine uptake, which all reduce the chances of severe illness, long-term health issues, or death from COVID-19. On the other hand, we also reviewed studies showing that fear of COVID-19 is related to psychological distress, panic buying, and xenophobia, and can have unequal effects across social groups. Taken together, the lessons learned during the COVID-19 pandemic can be used to prepare a more resilient response to the next pandemic.

## Transparency

*Action Editor:* Dolores Albarracin

*Editor:* Interim Editorial Panel

## Notes

1. *Self-efficacy* (i.e., one’s ability to perform responses to mitigate a threat) is often distinguished from *response efficacy* (i.e., the effectiveness of those responses; Ruiter et al., 2014). Here, for simplicity, we use “self-efficacy” to refer to both the appraisal of the effectiveness of responses to mitigate a threat and one’s ability to execute them.
